# Semiconductor fibers for textile integrated electronic systems

**DOI:** 10.1093/nsr/nwae143

**Published:** 2024-04-15

**Authors:** Yuanyuan Zheng, Zhen Wang, Peining Chen, Huisheng Peng

**Affiliations:** State Key Laboratory of Molecular Engineering of Polymers, Department of Macromolecular Science, Institute of Fiber Materials and Devices, and Laboratory of Advanced Materials, Fudan University, China; State Key Laboratory of Molecular Engineering of Polymers, Department of Macromolecular Science, Institute of Fiber Materials and Devices, and Laboratory of Advanced Materials, Fudan University, China; State Key Laboratory of Molecular Engineering of Polymers, Department of Macromolecular Science, Institute of Fiber Materials and Devices, and Laboratory of Advanced Materials, Fudan University, China; State Key Laboratory of Molecular Engineering of Polymers, Department of Macromolecular Science, Institute of Fiber Materials and Devices, and Laboratory of Advanced Materials, Fudan University, China

## Abstract

The near-room temperature resistance transition in the Lu-H-N compound is repeatedly reproduced, which is clarified to originate from a metal-to-semiconductor/insulator transition rather than superconductivity.

Over 5000 years of development, the functions of clothing have been mainly limited in warmth and modesty. With the development of smart fibers, fabrics are anticipated to revolutionize multidisciplinary applications, such as smart healthcare and the Internet of Things [1–4]. Presentation of the functions of smart fabric systems greatly relies on information processing by bulk chips and circuits, which brings challenges for wearability and stability due to the modulus mismatch. Flexible semiconductor fibers containing integrated semiconductor devices can be considered the last piece of the puzzle for fiber/textile electronic systems. However, the cooperative work of multiple devices for processing high-frequency signals presents requirements regarding the number and density of integrated units in a single fiber. In addition, the length of the fiber should be extended to accommodate the weaving process. Therefore, structure designs and preparation remain challenging because it is difficult to achieve the high performance and integration density of semiconductor devices on the limited and curved surfaces of fibers. This perspective mainly discusses the existing preparation technologies based on the above requirements. In addition, the future research directions are prospected at the end of the article.

To satisfy the demands for high performance and integration density for semiconductor fibers, the lithography and deposition stripping processes are recognized as the optimal routes for processing organic semiconductor materials such as pentacene (Fig. 1a). Traditional lithography technology typically involves coating a substrate with a light-sensitive photoresist, exposing it to ultraviolet (UV) light through a patterned mask, etching uncovered areas and stripping the photoresist to reveal a patterned substrate, which is mainly applicable to flat and rigid surfaces. To this end, a rotating lithography technology was developed to solve the processing issues of lithography on curved surfaces [5,6]. In a typical rotating lithography process, the outer surface of the cylindrical fiber is gradually scanned and patterned by a linear UV light source by controlling the axial rotation of the fiber. However, only a tiny proportion of functional devices can be integrated on the fiber surface because of the requirements for motion matching between the rotating motor and the light source. To solve this problem, methods of lithography on profiled fibers were further developed [7] in which the commercially advanced planar lithography technology is used to fabricate highly integrated devices on square or strip fibers. Ring oscillators are achieved by using this strategy. However, the flexibility and stability of the fibers still need to be carefully evaluated. Moreover, the integration level of the fiber makes it hard to meet application requirements, since at least 28 transistors are required to realize basic full-adder function for processing one-bit data.

**Figure 1.**
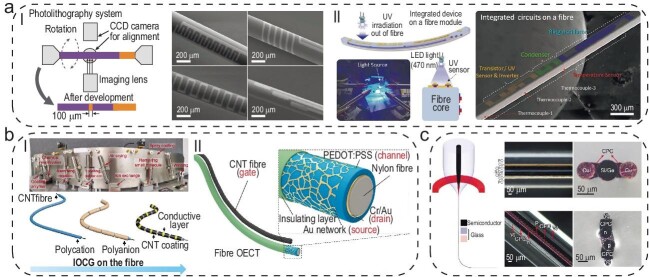
(a) Lithography technology for semiconductor fibers. (I) Rotating lithography process for curved surface on fiber [5,6]. (II) Methods of lithography on profiled fibers [7]. (b) Coating methods for flexible and long semiconductor fibers. (I) Industrial scrape-coating fabrication line for producing long fibers [9]. (II) Dip-coating functional layers sequentially on the fiber surface to realize organic electrochemical transistor with coaxial structure [8]. (c) Molten-core method producing fibers in hundreds of meters with single or multiple cores [10]. Figure 1a is reprinted with permission from Refs [5–7]. Figure 1b is reprinted with permission from Refs [8,9]. Figure 1c is reprinted with permission from Ref. [10].

In order to obtain continuous long semiconductor fibers, coating and/or molten-core methods have been developed. The coating method mainly applies functional layers sequentially on the fiber surface by scraping or dipping to realize a fiber with a coaxial structure for solution-processable semiconductor materials, such as conjugated polymers, quantum dots and metal oxide nanoparticles (Fig. 1b) [8,9]. However, it remains an unsolved problem to efficiently split the device along the fiber axis, so the achieved functions are mainly limited at the single-device level [8,9]. The molten-core methods have recently aroused great interest because they can produce inorganic fibers (based on Si and Ge) in hundreds of meters with single or multiple cores (Fig. 1c). In the molten-core process, the semiconductor core is melted into a fluid flow that is confined by the cladding and then thermally drawn into fibers [10]. Continuous and long semiconductor fibers with high optoelectronic properties have been demonstrated through rational mechanical designs [10] which are robust, weavable and waterproof. Although high-quality semiconductor fibers have been successfully fabricated by using the molten-core method, achieving complex structures that have the ability for efficient information processing remains challenging, such as different functional materials arranged and connected in a single fiber according to the logic circuit. It is difficult to precisely control temperature, material flow and connection to maintain the desired structural integrity and homogeneity, as the materials have different properties in axial and radial directions.

Semiconductor fibers with comparable flexibility to traditional textiles show broad applications in smart clothing, minimally invasive medical devices and robotics, although their performances, integration density and stability still have a considerable gap for practical applications. In order to further advance semiconductor fibers, the following directions should be carefully investigated as soon as possible.

Firstly, the properties of semiconductor fibers should be optimized through designs of new materials and advanced structures. Intrinsically flexible semiconductor fibers have advantages in stability and integration with traditional fibers. One effective strategy is to develop organic semiconductor materials with high electrical, optical and mechanical properties, such as conjugated polymers and small-molecule semiconductors. Besides, decreasing the diameter of inorganic semiconductor fibers will be helpful to strengthen their flexibility. Secondly, the integration level of semiconductor fibers needs to be greatly improved. Besides developing advanced micro–nano fabrication technologies suitable for the curved surface of fibers, another possible way is to increase the density of functional units along both the axial and radial directions. In addition, devices with memory functions such as memristors and latches can be integrated into the fiber to realize information storage and computing, which are the foundation of conventional devices such as computers and smartphones. Thirdly, the mass production process of semiconductor fibers requires to be improved. Engineered fiber lithography production lines with increased length will enable the production of longer semiconductor fibers while ensuring device integration and performance. Finally, it is necessary to enhance the stability of semiconductor fibers during use and post-processing. Effective fiber mechanical designs can greatly enhance functional stability, such as island and soft–hard segment structure designs. Besides, the development of packaging processes, such as fluorinated polymer coating and parylene meteorological deposition, can facilitate adaptation to various environmental conditions such as wear and wash.

Future semiconductor fibers should be flexible, highly integrated, multifunctional, mass-productive and stable. They should achieve the functions of data reception, processing and transmission, and be able to be stably woven into intelligent full-textile electronic systems without bulk chips and circuits. We believe that this vision will come to reality soon with the efforts of researchers.
